# Effect of glycosylation on the affinity of the MTB protein Ag85B for specific antibodies: towards the design of a dual-acting vaccine against tuberculosis

**DOI:** 10.1186/s13062-024-00454-5

**Published:** 2024-01-25

**Authors:** Roberta Bernardini, Sara Tengattini, Zhihao Li, Luciano Piubelli, Teodora Bavaro, Anamaria Bianca Modolea, Maurizio Mattei, Paola Conti, Stefano Marini, Yongmin Zhang, Loredano Pollegioni, Caterina Temporini, Marco Terreni

**Affiliations:** 1 Department of Translational Medicine, University of Tor Vergata, Via Montpellier 1, Rome, 00133 Italy; 2 Interdepartmental Center for Comparative Medicine, Alternative Techniques and Aquaculture (CIMETA), University of Rome “Tor Vergata”, Via Montpellier 1, Rome, 00133 Italy; 3 Drug Sciences Department, University of Pavia, Viale Taramelli 12, Pavia, 27100 Italy; 4 Parisian Institute of Molecular Chemistry, Sorbonne University, UMR CNRS 8232, 4 Place Jussieu, Paris, 75005 France; 5 Department of Biotechnology and Life Sciences, University of Insubria, via J.H. Dunant, 3, Insubria, Varese 21100 Italy; 6 Department of Biology, University of Rome “Tor Vergata”, Rome, Italy; 7 Department of Pharmaceutical Sciences, University of Milan, Via Mangiagalli 25, Milan, 20133 Italy

**Keywords:** Lipoarabinomannan, Tuberculosis, *Mycobacterium*, Glycoconjugate, Antibodies, Vaccine, Immune response

## Abstract

**Background:**

To create a dual-acting vaccine that can fight against tuberculosis, we combined antigenic arabino-mannan analogues with the Ag85B protein. To start the process, we studied the impact of modifying different parts of the Ag85B protein on its ability to be recognized by antibodies.

**Results:**

Through our research, we discovered that three modified versions of the protein, rAg85B-K30R, rAg85B-K282R, and rAg85B-K30R/K282R, retained their antibody reactivity in healthy individuals and those with tuberculosis. To further test the specificity of the sugar AraMan for AraMan antibodies, we used Human Serum Albumin glycosylated with AraMan-IME and Ara_3_Man-IME. Our findings showed that this specific sugar was fully and specifically modified. Bio-panning experiments revealed that patients with active tuberculosis exhibited a higher antibody response to Ara_3_Man, a sugar found in lipoarabinomannan (LAM), which is a major component of the mycobacterial cell wall. Bio-panning with anti-LAM plates could eliminate this increased response, suggesting that the enhanced Ara_3_Man response was primarily driven by antibodies targeting LAM. These findings highlight the importance of Ara_3_Man as an immunodominant epitope in LAM and support its role in eliciting protective immunity against tuberculosis. Further studies evaluated the effects of glycosylation on the antibody affinity of recombinant Ag85B and its variants. The results indicated that rAg85B-K30R/K282R, when conjugated with Ara_3_Man-IME, demonstrated enhanced antibody recognition compared to unconjugated or non-glycosylated versions.

**Conclusions:**

Coupling Ara_3_Man to rAg85B-K30R/K282R could lead to the development of effective dual-acting vaccines against tuberculosis, stimulating protective antibodies against both AraMan and Ag85B, two key tuberculosis antigens.

## Introduction

Tuberculosis (TB) is a significant global health challenge, with over 10 million new cases and 1.5 million deaths reported annually [1]. Drug resistance is a major obstacle to TB control. Multi-drug-resistant TB (MDR-TB) represents the pathological condition resistant to both isoniazid and rifampicin, the two most potent first-line anti-TB drugs. Extensively drug-resistant TB (XDR-TB) is even more resilient, with resistance to at least three of the six most effective anti-TB drugs [2]. Numerous studies have been conducted on the protein domains involved in both host and pathogen proteins involved in the infection mechanism [3] and host-directed therapies (HDTs) are a new approach to TB treatment that targets the host’s immune system to help it fight the infection. HDTs have shown promise in early clinical trials, but they are still in development [4, 5]. Vaccines are the most effective way to prevent infectious diseases, including TB. However, the only licensed TB vaccine, Bacille Calmette-Guérin (BCG), is not effective enough in preventing TB disease in adults. BCG is more effective in preventing TB meningitis in children, but it does not provide long-term protection against pulmonary TB [6, 7].

Extensive research has been carried out on non-natural glycoproteins and glycolipids to develop new therapeutic approaches for infectious diseases and cancer [8, 9]. Carbohydrate-based vaccines have been investigated as a potential treatment option [10–13]. To create anti-infective vaccines, antigenic oligosaccharides naturally present on the pathogen’s membrane are chemically conjugated with specific immunogenic carrier proteins like diphtheria toxoid, tetanus toxoid, and cross-reactive material 197 (a mutant form of diphtheria toxoid) [14, 15]. The use of natural oligosaccharides obtained from pathogens allowed the development of effective vaccines against different serotypes of *Haemophilus influenzae* type b (Hib) virus and *Streptococcus pneumoniae*, or *Neisseria meningitidis* [16] and more recently Varicella-zoster virus [17]. Synthetic oligosaccharides have been used as an alternative for the preparation of a vaccine against Hib [18] to avoid the problems related to the production of pure oligosaccharides from natural sources.

Arabinomannan (AM) is the major carbohydrate antigen of MTB and is part of the lipoarabinomannan (LAM) membrane glycolipids. This oligosaccharide has been considered for the development of new glycovaccines against TB. However, it is very complex and difficult to be prepared by total synthesis. For this reason, synthetic analogues have been recently investigated as antigens for the development of carbohydrate-based TB vaccines [19–22].

New synthetic strategies are continuously proposed to reduce the drawbacks of the chemical synthesis of oligosaccharides. In this context, biocatalysis is considered an important tool to reduce the synthetic steps [23]. In past years, we have developed enzymatic strategies for the synthesis of acetylated sugar building blocks with free hydroxyl groups at desired position [24, 25] that can be then chemically assembled to obtain complex oligosaccharides using the acetyl as the only protecting group [26]. This approach has been recently employed for the chemoenzymatic synthesis of antigenic AM analogues composed by the combination of different units of mannose and arabinose [19].

On the other hand, subunit vaccines obtained by gene fusion of different antigenic TB proteins are under investigation. Ag85B, the major protein antigen of MTB, has been considered for the development of several subunit vaccines that have been submitted to clinical investigation, aiming to develop efficient vaccines alternative to the BCG [27].

Ag85B was also conjugated to natural AM isolated from MTB, to obtain vaccines with high antigenic properties prepared by combining two different MTB antigens [12, 28, 29]. However, the biological activity of neo-glycoproteins based on antigenic proteins could be negatively affected by the glycosylation of the protein epitopes [30, 31]. In fact, the T-cell activity of Ag85B was strongly depressed by chemical glycosylation targeting lysines (the most used approach for the preparation of neo-glycoproteins) regardless the kind of sugar used [30]. Several works dealing with the characterization of the T-cell epitopes of this protein have been published [30, 32]. Still, only a few of them contained data on the characterization of B-cell epitopes recognized by human antibodies [30, 31]. A detrimental effect induced by chemical glycosylation of recombinant Ag85B (rAg85B) on T-cell activity was observed and ascribed to the modification of two lysines involved in relevant epitopes [33]. Accordingly, rAg85B variants obtained by replacement of these lysines with arginines have been designed to avoid their glycosylation. Thus, rAg85B, which bears seven additional amino acid residues at the N-terminal region with respect of the sequence of the wild-type (wt) Ag85B [34], was compared with its variants obtained by Lys (K) to Arg (R) substitution at position 30 and 282 (corresponding to K23 and K275, respectively). These rAg85B variants maintained the T-cell activity after glycosylation unlike the wild-type rAg85B [33].

Little is known about the effect of glycosylation on the recognition of antigenic protein by human antibodies. Therefore, in order to develop a dual-acting vaccine obtained by conjugation of antigenic AM analogues with Ag85B, here we evaluated the effect induced by glycosylation of rAg85B on the affinity for antibodies present in the serum of TB patients. This work reports the preparation of different neo-glycoproteins using sugars activated with an iminomethoxyethyl (IME) reactive linker and different proteins. The different products were evaluated in the binding of antibodies obtained from patients with active TB and from healthy controls.

## Materials and methods

### Production of proteins and neo-glycoproteins

Recombinant Ag85B (rAg85B) and its variants (obtained by substitution of a Lys residue with Arg at position 30 and/or 282) were prepared according to the previously reported methods [33, 34]. This approach yields proteins with seven additional amino acid residues at the N-terminal compared to the sequence of the wt Ag85B. Thus, positions 30 and 282 in rAg85B correspond to positions 23 and 275 in the sequence of the wt protein.

The synthesis of the different glycans activated by an IME linker [19, 35],the chemical glycosylation of proteins, and the characterization of the neo-glycoproteins obtained were carried out according to the procedures previously reported for the different supports: commercial recombinant human serum albumin (rHSA, Oryzogen, Wuhan, China) [19], rAg85B [30] and variants [33].

### Studied population

The study enrolled 47 individuals with newly diagnosed and untreated active pulmonary tuberculosis, along with 40 healthy individuals with no history of tuberculosis exposure (referred to as TB unexposed controls). In all cases, the diagnosis of active TB was confirmed through *M. tuberculosis* culture isolation.

### ELISA assay

Antibodies against Ag85B proteins and their glycoderivatives were detected using an ELISA assay with minor modifications based on a previously described protocol [36].

The ELISA assay was performed in 96-well microplates (Nunc Maxisorb, Becton Dickinson). The antigens (recombinant proteins: rAg85B, rAg85B-K30R, rAg85B-K282R, rAg85B-K30R/K282R, synthesized oligosaccharides: AraMan, Ara_3_Man, Man_2_ and the conjugated glycoproteins: rAg85B + Man_2_, rAg85B-K30R/K282R + Man_2_, rAg85B-K30R/K282R + Ara_3_Man).

were diluted to a final concentration of 10 µg/mL in 100 µL of carbonate buffer (pH 9.6) and incubated for 2 h at room temperature followed by overnight incubation at 4 °C. The wells were then washed twice with phosphate-buffered saline (PBS) containing 0.05% Tween-20 (PBS-T) to remove any unbound proteins.

To block the non-specific binding sites, the wells were incubated (37 °C, 1 h) with PBS-T containing 3% bovine serum albumin (BSA). Subsequently, the plates were incubated for 1 h at room temperature with 100 µL of serum diluted 1:100.

For the ELISA panning assay. plates were coated with lipoarabinomannan (LAM BEI Resources, NIAID, USA) diluted (final concentration 2 µg/mL) in 100 µL of carbonate buffer (pH 9.6), then incubated, washed and blocked using the same procedure described before. The sera were incubated (RT, 1 h) with 100 µL of serum (1:100), and the procedure was repeated three times (in three different plates subsequently), keeping all other conditions identical as before. After the incubation with sera, all the plates (those analyzed with the antigens and those analyzed with LAM for the panning) were incubated (RT, 1 h) with anti-human IgG Secondary Antibody HRP conjugated (Invitrogen, Italy). The absorbance (OD) at 492 nm of antigen- and buffer-coated wells was measured, and the difference in mean OD values was calculated. All samples were assayed in duplicate to increase precision.

### Statistical analysis

Data were reported as the mean values ± standard error of the mean (SEM) or standard deviation (SD). Statistical comparisons between two groups were performed by Student’s t-test. *p* values < 0.05 were considered to be statistically significant. GraphPad Prism version 5.0 (GraphPad Software, Inc., La Jolla, CA) was used for all statistical analyses and graphs.

## Results

### Ag85B variants preserved the antibody reactivity in healthy and TB human samples

**Fig. 1:**
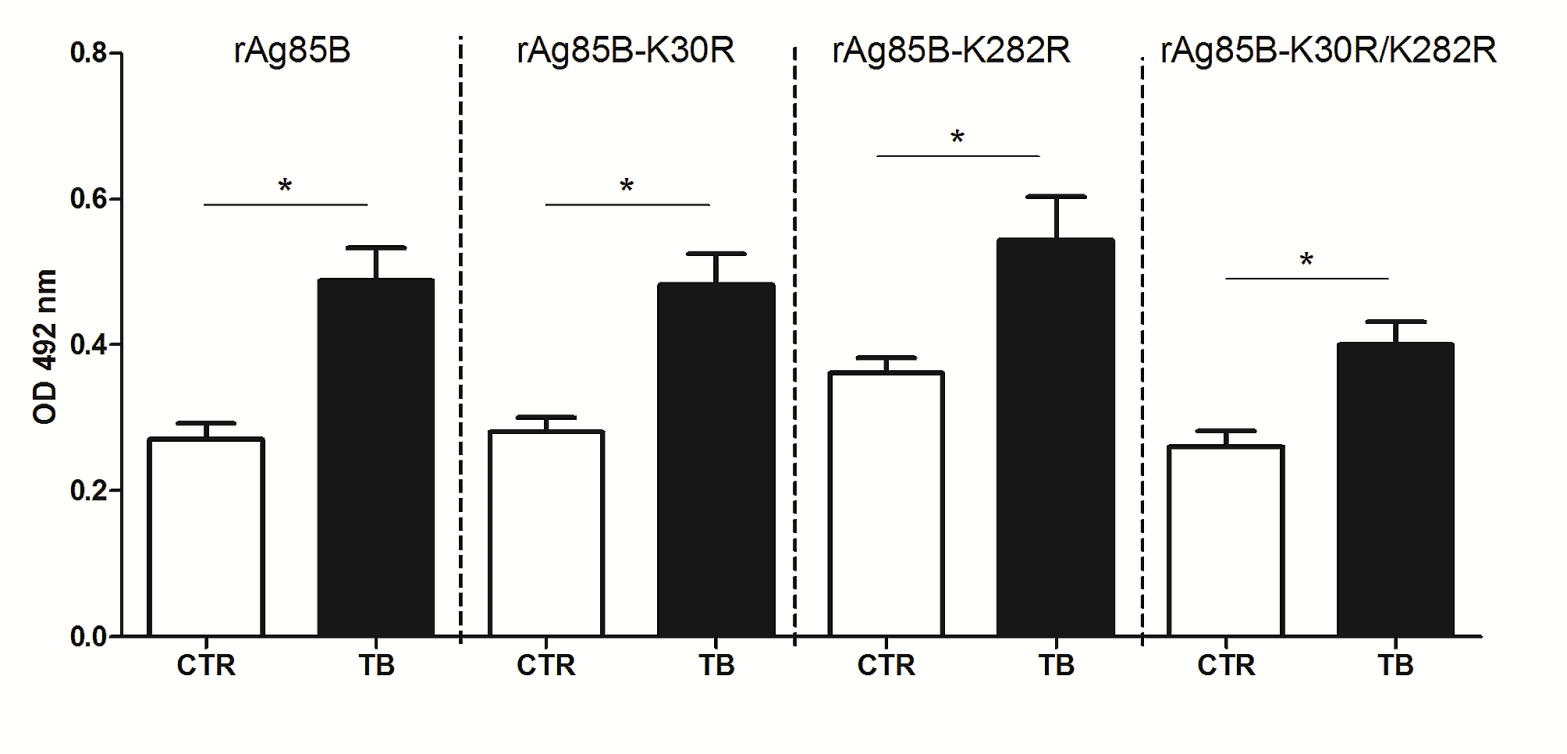
Levels of antibodies (IgG) reactive against the rAg85B and its variants in TB patients (TB: 47 samples) and healthy subjects (CTR: 40 samples). All samples were measured in duplicate and repeated three times. The data are presented as mean values ± standard error of the mean (SEM). OD = optical density; *=*p* ≤ 0.05 by Student’s t-test

To evaluate the impact of substitutions of Lys30 and/or Lys282 of Ag85B with arginine (rAg85B-K30R, rAg85B-K282R and rAg85B-K30R/K282R, respectively), sera from control and TB subjects were investigated. Control sera indicated comparable antibody levels against rAg85B and its variants (Fig. 1), except a slightly higher value for the K282R variant. In TB patients, significantly increased antibody levels were detected for rAg85B and its variants, compared to controls. This enhancement in TB patients was uniform for each antibody (+ 50–80%) relative to each control; no significant difference was observed between rAg85B and its variants in TB patients. This analysis demonstrates that, while antibody levels against the different rAg85B variants are increased in TB *vs* controls, no significant differences can be detected when normal subjects were compared. Therefore, the introduced substitutions do not alter the antibody response.

### Preparation and characterization of neo-glycoproteins

**Fig. 2:**
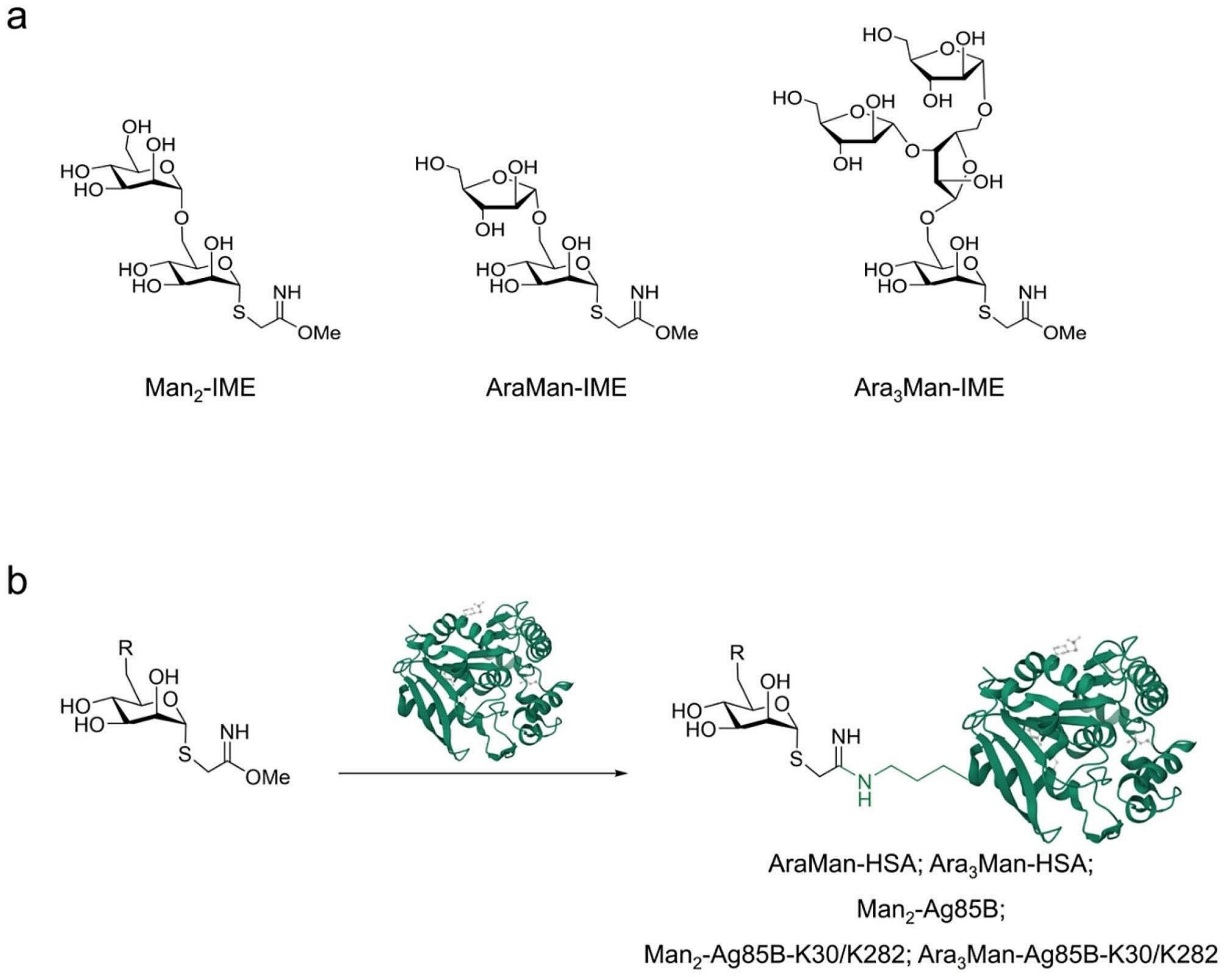
**(a)** Chemical structure of the different glycans activated with IME linker. **(b)** Chemical glycosylation of proteins. Reactions were performed in 100 mM sodium tetraborate pH 9.5 at 25 °C for 24 h; glycosidic reagent/protein molar ratio: 250:1 for Ara_3_Man-rAg85B-K30R/K282R, and 200:1 for the others; protein concentration: 2 mg/mL. In rAg85B and rAg85-K30R/K282R glycosylation, 1mM benzamide hydrochloride was added in the reaction mixture to avoid potential proteolysis [33]. Protein graphical representation depicts Ag85B protein and was taken from the Protein data Bank (PDB DOI: 10.2210/pdb1f0p/pdb)

The Man_2_, AraMan and Ara_3_Man glycans, activated in an anomeric position with the IME reactive linker (Fig. 2a), which selectively targets the amino group of lysine residues [35], were used to prepare the different neo-glycoproteins (Fig. 2b) considered in the evaluation of antibody affinity.

After conjugation, the degree of glycosylation of the resulting neo-glycoproteins was determined by analyzing the residual presence of unmodified protein and the average glycan loading.

For rHSA derivatives, conjugation was assessed by hydrophilic interaction liquid chromatography (HILIC) and high-resolution mass spectrometry (HRMS), as previously described [19]. Both samples, AraMan and Ara_3_Man, showed the complete modification of rHSA and an average loading of 9.3 and 14.5 [19] mol/mol, respectively.

For Man_2_-rAg85B the conjugation was quantitative, with no residual presence of unmodified protein and an average loading of 4.8 disaccharide units *per* protein. The substitution of lysine 30 and 282 significantly reduced protein reactivity, as previously described [33], yielding to a 98% glycosylation degree and an average loading of 2.5 mol/mol. To compensate the further reduction of reactivity due to the increased saccharide size, the glycan/protein molar ratio was increased to 250:1 in the preparation of Ara_3_Man-Ag85B-K30R/K282R. This resulted in 100% glycosylation yield and an average loading of 2.8 mol/mol.

### The LAM-specific antibodies were subtracted by three rounds of bio-panning

To characterize the antibody reactivity to the AM mimetic sugar antigen, the AraMan and Ara_3_Man motifs were examinated after conjugation with HSA. Control and TB sera were placed into wells containing a non-immunogenic protein (HSA) or the same protein glycosylated with the disaccharide (AraMan-rHSA) or the tetrasaccharide (Ara_3_Man-rHSA). To determine whether antibodies against these motifs were specific for LAM, negative affinity purification of antibodies was also performed. In detail, samples of sera were placed into wells containing LAM for three rounds of subtractive (negative) panning in vitro. Sera negativity to rHSA was tested by ELISA in the same experiment.

**Fig. 3:**
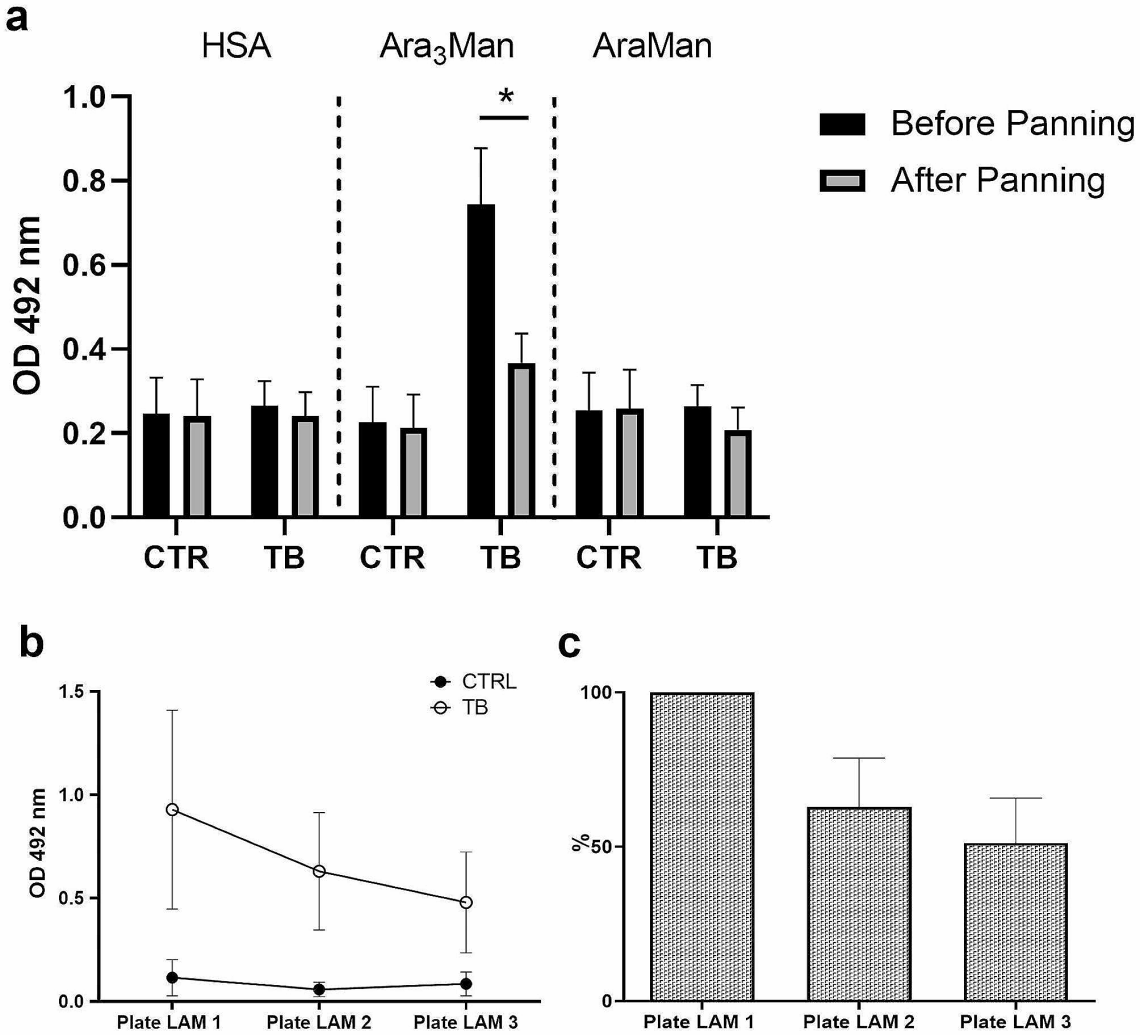
ELISA analysis. **(a)** ELISA analysis for the determination of antibody (IgG) levels against HSA, Ara_3_Man-rHSA and AraMan-rHSA was carried out using samples obtained before and after three rounds of selection against LAM in healthy subjects (CTR) and in TB patients. Absorbance values are the mean of 9 control samples and 11 TB samples in each group. All samples were measured in duplicate and repeated three times. Error bars show standard error of the mean (SEM) for each set of data. *=*p* ≤ 0.05 by Student’s t-test. The level of antibodies against Ara_3_Man is very high in TB patients, and is reduced by about 50% in TB patients when LAM post-adsorption serum was tested. **(b, c)** Detection of anti-LAM antibody level upon 3 bio-panning procedures. The level of specific antibodies against LAM was measured after each bio-panning step. The same sera were tested on three adsorbed LAM plates consecutively (from Plate LAM 1 to Plate LAM 3); the reduction is expressed in OD mean values ± standard error of the mean (SEM) **(b)** and in percentage **(c)** of healthy control and TB subjects **(b)** or only TB **(c)**, respectively. High levels of anti-LAM antibodies are detected in TB patients on bio-panning steps (reduced by about 50% from step 1 to step 3), while no anti-LAM antibodies could be found in healthy controls

Before the panning, the ELISA test showed a higher Ara_3_Man antibodies level in TB subjects compared to experiments performed with the non-glycosylated protein and with the disaccharide AraMan (*p* < 0.0037 and *p* < 0.0031 respectively) (Fig. 3). However, this difference disappeared after three rounds of bio-panning; the reduction in the response against Ara_3_Man was statistically significant (*p* < 0.0215) compared to the levels obtained before the bio-panning. These results indicate that the increase in Ara_3_Man response level was mainly caused by LAM-specific antibodies.

To highlight the anti-LAM antibody level present in the sera, LAM-coated plates were set up for bio-panning. Figure 3 shows the levels of anti-LAM antibodies expressed as OD (panel b) or percentage relative to Plate LAM 1 (panel c) in the three different plates used to purify the sera. There is a decrease in anti-LAM antibodies in each bio-panning step for TB patients. This indicates that some of the LAM-specific antibodies are removed from the sera of TB patients using LAM purified from *M. tuberculosis.* The experiments also show that Ara_3_Man is a motif contained in the LAM.

### Effects of glycosylation on antibody reactivity

**Fig. 4:**
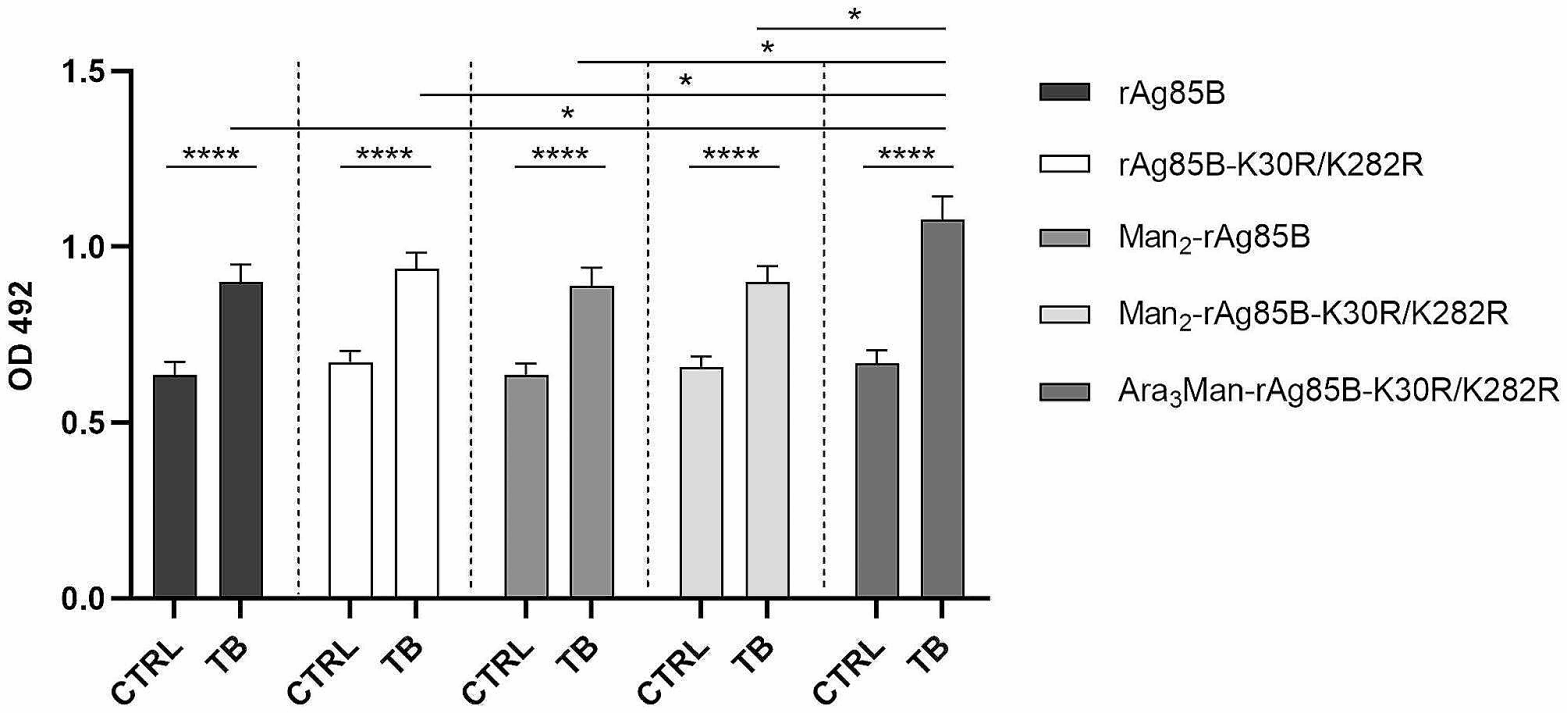
Distribution of antibodies (IgG) levels reactive with rAg85B, its double variant and the corresponding glycoderivatives, in TB patients and healthy subjects (14 TB samples and 17 controls). All samples were measured in duplicate and repeated three times. The data are presented as mean values ± standard error of the mean (SEM). OD = optical density; CTR: controls; TB = tuberculosis. *p* ≤ 0.05 = *, *p* ≤ 0.0001 = **** were calculated by Student’s t-test

rAg85B and the rAg85B-K30R/K282R variant proteins were glycosylated to evaluate the effect of the conjugation with the di- or tetrasaccharide on the antibody affinity of the different glycoproteins. The experiments were performed by ELISA test on sera from healthy controls and TB subjects. The results showed that there was a statistically significant difference between TB samples and their controls for rAg85B and its double variant. However, healthy subjects did not show increased antibody reactivity to all glycosylated proteins. Notably, TB subjects show a statistically significant increase in affinity for Ara_3_Man-rAg85B-K30R/K282R when compared with all TB groups (*p* ≤ 0,0217 vs. rAg85B; *p* ≤ 0,0513 vs. rAg85B-K30R/K282R; *p* ≤ 0,0173 vs. Man_2_-rAg85B; *p* ≤ 0,0178 vs. Man_2_- rAg85B-K30R/K282R) (Fig. 4).

Therefore, TB subjects have the highest levels of antibodies with reactivity towards the rAg85B-K30R/K282R variant conjugated with Ara_3_Man, due to the synergistic effect of the two antigens (protein and sugar moiety), which may provide the basis for a dual-acting vaccine.

## Discussion

In this study, we first analyzed the effect of selected Ag85B modification on antibody recognition. Then, Human Serum Albumin (HSA) glycosylated with AraMan-IME and Ara_3_Man-IME was considered to assess the specificity of the last sugar for AM antibodies.

In addition, rAg85B and its variant obtained by K/R substitution at positions 30 and 282 (rAg85BK30R/K282R) and glycosylated with Man_2_-IME was studied to assess the effect of the glycosylation on the antibody affinity of these antigenic proteins. Finally, conjugation of rAg85B-K30R/K282R double variant with Ara_3_Man-IME has been investigated as a proof of concept about the potential development of dual-acting vaccines against TB targeting antibodies specific for two different types of MTB antigens (the AM sugar and the Ag85B protein).

Given the success of several carbohydrate-based anti-bacterial vaccines, including those against facultative intracellular organisms [37–39], we focused our efforts on constructing a carbohydrate-protein conjugate vaccine against TB using an oligosaccharide that mimics the LAM antigen of MTB. For this purpose, Ag85B was selected as the carrier protein in order to design a double-acting vaccine, since it is one of the most potent antigenic species expressed by MTB.

Therefore, a recombinant form of Ag85B (rAg85B) [34] and the K30R and/or K282R variants were prepared. The substitution of these amino acids, which are involved in the main T-cell epitopes of Ag85B, proved to be conservative for protein conformation: ex-vivo ELISPOT experiments demonstrated that all protein variants maintained the original T-cell immunogenic activity exhibited by rAg85B [33]. The activity of the Ag85B variants was also maintained after glycosylation, unlike the wild-type protein, since the introduced substitutions avoid glycosylation of the main T-cell epitopes [33]. In the present work, to complete the evaluation of the immunological response to these recombinant proteins, antibody recognition of wt rAg85B and of its variants was investigated. In an ELISA test, all recombinant proteins showed similar efficiency of recognition of antibodies present in the serum of MTB-infected patients (Fig. 1). In addition, glycosylation with non-antigenic disaccharide (Man_2_ and AraMan) had little effect on the immuno-reactivity of these proteins, confirming them as putative antigenic carriers functional for the design of effective glycoconjugated dual-acting vaccines against MTB (Figs. 3a and 4) using a double hit approach (combining sugar and protein antigens).

LAM has been extensively studied for its immunomodulatory properties and as a structurally unique glycolipid component of the envelope of all mycobacterial species [40] and is therefore considered an attractive vaccine candidate [41, 42] to evoke immune responses against MTB. Anti-LAM antibodies are induced during MTB infection [43–45] and have been associated with bacterial opsonization and restriction of intracellular growth [46, 47].

Pure oligosaccharides are poor immunogens as they fail to recruit CD4^+^ T cell help. They are therefore limited to T cell–independent B cell immune responses. However, conjugating a bacterial polysaccharide to an immunogenic carrier protein that provides T-cell epitopes creates a T cell–dependent antigen that can induce protective immunity. Indeed, AM isolated from LAM derived from the MTB cell wall and conjugated to various immunogenic carrier proteins has been used to generate new glyco-conjugate TB vaccine candidates [48]. However, AM-conjugated products using different vaccination protocols showed modest protection, never exceeding the effect of BCG vaccination [28, 29, 49–51]. In addition, natural AM is too complex for chemical synthesis to develop semi-synthetic glyco-vaccines. For this reason, we studied the synthesis of AM analogues structurally related to the natural MTB antigen, which allowed the synthesis of the Ara_3_Man that showed affinity to LAM antibodies of infected patients [19].

In the present work, we have demonstrated the specificity of this oligosaccharide for the LAM antibodies of TB patients and its synergic activity towards human TB-antibodies after conjugation with a variant of rAg85B. Actually, ELISA experiments showed that the increased affinity induced by Ara_3_Man after conjugation with HSA (Fig. 3) was caused by LAM-specific antibodies. To further validate this specificity, LAM-specific antibodies were selectively removed from TB patient sera using purified LAM from *M. tuberculosis* (Fig. 3). This depletion of LAM-specific antibodies led to a significant decrease in immune recognition of TB samples, confirming the strong correlation between LAM and the Ara_3_Man motif.

In addition, the immunogenic activity of rAg85B-K30R/K282R variant conjugated with different synthetic oligosaccharides was investigated. Glycosylation with non-antigenic disaccharides maintained the antibody affinity of the Ag85B variant protein, while conjugation with Ara_3_Man tetrasaccharide enhanced it. This improvement was likely due to an additional interaction with LAM-specific antibodies (Fig. 4).

ELISA experiments revealed that TB patients had significantly higher levels of antibodies against the rAg85B-K30R/K282R variant conjugated with Ara_3_Man compared to the non-conjugated protein or the one conjugated with Man_2_, a non-antigenic disaccharide. These findings suggest that Ara_3_Man could serve as a promising glycan component for developing more effective glycoconjugate TB vaccines. By combining Ara_3_Man with rAg85B or other relevant antigens, a synergistic effect could be achieved, potentially leading to enhanced immune responses and improved protection against TB. For this reason, the results provide a proof-of-concept for the development of a dual-acting vaccine that targets two different MTB antigens.

The next steps will involve evaluating the immunogenicity of this conjugate in animal models and assessing its protective efficacy after the challenge. Additionally, further investigations will explore alternative AM-mimetic oligosaccharides to optimize the immune response mediated by the sugar antigen.

## Data Availability

The datasets used and/or analysed during the current study are available from the corresponding author on reasonable request.
